# Prediction of overall survival in patients with pancreatic ductal adenocarcinoma: histogram analysis of ADC value and correlation with pathological intratumoral necrosis

**DOI:** 10.1186/s12880-022-00751-3

**Published:** 2022-02-08

**Authors:** Yoshifumi Noda, Hiroyuki Tomita, Takuma Ishihara, Yoshiki Tsuboi, Nobuyuki Kawai, Masaya Kawaguchi, Tetsuro Kaga, Fuminori Hyodo, Akira Hara, Avinash R. Kambadakone, Masayuki Matsuo

**Affiliations:** 1 Department of Radiology, Gifu University, 1-1 Yanagido, Gifu, 501-1194 Japan; 2 Department of Tumor Pathology, Gifu University, 1-1 Yanagido, Gifu, 501-1194 Japan; 3 Innovative and Clinical Research Promotion Center, Gifu University Hospital, 1-1 Yanagido, Gifu, 501-1194 Japan; 4 Department of Radiology, Frontier Science for Imaging, Gifu University, 1-1 Yanagido, Gifu, 501-1194 Japan; 5 Department of Radiology, Massachusetts General Hospital, Harvard Medical School, 55 Fruit Street, White 270, Boston, MA 02114 USA

**Keywords:** Diffusion magnetic resonance imaging, Pancreatic cancer, Prognosis

## Abstract

**Background:**

To evaluate the utility of histogram analysis (HA) of apparent diffusion coefficient (ADC) values to predict the overall survival (OS) in patients with pancreatic ductal adenocarcinoma (PDAC) and to correlate with pathologically evaluated massive intratumoral necrosis (MITN).

**Materials and methods:**

Thirty-nine patients were included in this retrospective study with surgically resected PDAC who underwent preoperative magnetic resonance imaging. Twelve patients received neoadjuvant chemotherapy. HA on the ADC maps were performed to obtain the tumor HA parameters. Using Cox proportional regression analysis adjusted for age, time-dependent receiver-operating-characteristic (ROC) curve analysis, and Kaplan–Meier estimation, we evaluated the association between HA parameters and OS. The association between prognostic factors and pathologically confirmed MITN was assessed by logistic regression analysis.

**Results:**

The median OS was 19.9 months. The kurtosis (*P* < 0.001), entropy (*P* = 0.013), and energy (*P* = 0.04) were significantly associated with OS. The kurtosis had the highest area under the ROC curve (AUC) for predicting 3-year survival (AUC 0.824) among these three parameters. Between the kurtosis and MITN, the logistic regression model revealed a positive correlation (*P* = 0.045). Lower survival rates occurred in patients with high kurtosis (cutoff value > 2.45) than those with low kurtosis (≤ 2.45) (*P* < 0.001: 1-year survival rate, 75.2% versus 100%: 3-year survival rate, 14.7% versus 100%).

**Conclusions:**

HA derived kurtosis obtained from tumor ADC maps might be a potential imaging biomarker for predicting the presence of MITN and OS in patients with PDAC.

## Introduction

Pancreatic ductal adenocarcinoma (PDAC) is a lethal cancer with a 5-year survival rate of only 6% [1]. Surgical resection offers curative remedy; however, only 20% of patients have a resectable tumor at the time of diagnosis. In patients underwent surgical resection, rapid recurrence results in a 5-year relative survival of 39% [2]. Neoadjuvant chemotherapy or chemoradiation therapy has been increasingly used in recent years in the preoperative management of patients with borderline resectable or locally advanced PDAC. This has enabled curative surgical resection in a larger cohort of patients [3]. On the other hand, a substantial proportion of patients develop early recurrence or distant metastasis after resection. Therefore, it is important to identify preoperative prognostic factors to determine long term outcome.

Magnetic resonance imaging (MRI) is the preferred imaging modality for the evaluation of liver metastases in patients with PDAC [4]. Apparent diffusion coefficient (ADC) measured with diffusion-weighted imaging allows non-invasive assessment of water diffusion reflecting tissue microstructure [5]. In recent years, ADC histogram analysis (HA) has been investigated as a quantitative imaging biomarker to depict the distribution and frequency of ADC values and therefore determine heterogeneity of water diffusivity within the tumor [6, 7]. This technique has been applied for determining tumor aggressiveness and predicting tumor recurrence in various malignancies such as uterine cervical cancer [8], bladder cancer [9], rectal cancer [10], pancreatic intraductal papillary mucinous neoplasms [11], and pancreatic neuroendocrine tumor [12]. These studies have revealed that certain HA parameters such as kurtosis, 10th percentile, entropy, or skewness could predict pathological complete response or tumor grade. Although, HA on dual-energy CT was used for assessing chemotherapeutic response in patients with PDAC [13], we could not find any literature that performed ADC HA of PDAC.

In patients with PDAC, various imaging features such as hypodensity on CT image at portal venous phase [14], irregular tumor margin [15], and rim enhancement [16] have been reported to be associated with poor prognosis in previous studies. Intratumoral necrosis which is associated with these imaging findings has been reported as a predictor of poor prognosis after surgical resection [17]. Massive intratumoral necrosis (MITN) has been associated with the imaging appearance of rim enhancement with central hypodensity. We hypothesized that HA of ADC maps could be used to determine presence of MITN and therefore help predict patient outcome. Therefore, we undertook this study to evaluate the use of ADC HA to predict the overall survival (OS) and correlate with MITN in patients with PDAC.

## Materials and methods

### Patients

**Fig. 1:**
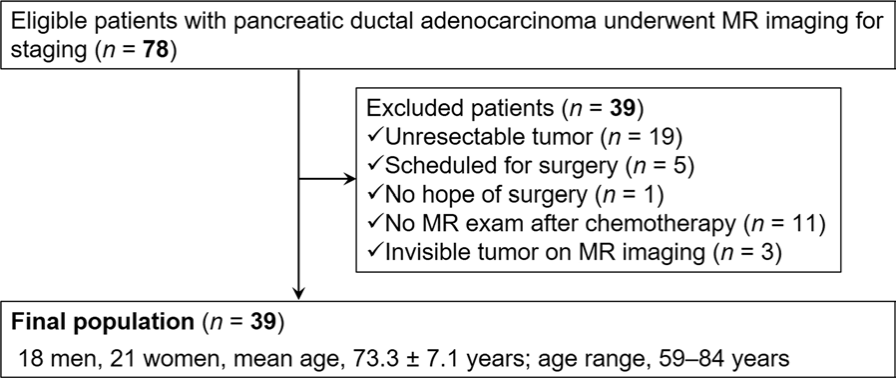
Flow chart of included and excluded patients

Seventy-eight consecutive patients with known PDAC based on a previously performed endoscopic ultrasound-guided fine-needle aspiration underwent MRI between February 2015 and March 2020 were included. Thirty-nine out of 78 patients were excluded due to: lack of pathological proof (*n* = 25) (locally advanced tumor or having distant metastasis [*n* = 19], scheduled for surgery [*n* = 5], and rejection of surgery [*n* = 1]), absence of MRI after neoadjuvant chemotherapy (*n* = 11), or inability to visualize tumor on MR images (*n* = 3). The final cohort included 39 patients (mean age, 73.3 ± 7.1 years; age range, 59–84 years) including 18 men (mean age, 74.3 ± 6.4 years; age range, 63–84 years) and 21 women (mean age, 72.5 ± 7.8 years; age range, 59–83 years) (Fig. 1).

Out of the 39 patients, 30.8% of patients (*n* = 12/39) received preoperative neoadjuvant chemotherapy, and 69.2% (*n* = 27/39) patients underwent upfront surgical resection without neoadjuvant treatment. The neoadjuvant chemotherapeutic regime included: combined gemcitabine and S–1 (*n* = 6), combined gemcitabine and nab-paclitaxel (PTX) (*n* = 4), or FOLFIRINOX (folinic acid, fluorouracil, irinotecan, and oxaliplatin) (*n* = 2). Pancreaticoduodenectomy with pancreaticojejunostomy reconstruction was performed in 53.8% (*n* = 21) and distal pancreatectomy in 46.2% (*n* = 18). The mean interval between preoperative MRI and pancreatic surgery was 21.8 days (range: 3–81 days). The patient’s medical records were reviewed to document patients’ demographics, plasmatic carcinoembryonic antigen (CEA) and carbohydrate antigen (CA) 19–9 levels, treatment details, and surgical and pathology reports.

### MRI parameters

MRI of the pancreas was performed on a 3 T MR system (Intera Achieva Quasar Dual or Ingenia 3.0 T CX; Philips Medical Systems, Best, the Netherlands) equipped with a 32-channel digital coil. The MRI protocols included the following sequences: three-dimensional fat-suppressed axial T1-weighted fast field echo imaging; in-phase and opposed-phase T1-weighted axial gradient-recalled-echo imaging; respiratory-triggered two-dimensional fat-suppressed axial T2-weighted turbo spin-echo imaging (using Multi Vane in Ingenia 3.0T CX); and free-breathing two-dimensional axial diffusion-weighted imaging with a single-shot echo-planar sequence (Table 1).

**Table 1:** MRI sequences and parameters

Parameter	Intera Achieva Quasar Dual				Ingenia 3.0T CX			
	T1WI	In/out	T2WI	DWI	T1WI	In/out	T2WI	DWI
Sequence	3D TFE	GRE	2D TSE	2D EP	3D TFE	GRE	2D TSE MV	2D EP
Respiratory control	BH	BH	RT	FB	BH	BH	RT	FB
Fat suppression	Yes	No	Yes	Yes	Yes	No	Yes	Yes
TR (ms)	3.3	242	1900	5000	3.3	223	2000	5000
TE (ms)	1.57	1.2/2.4/3.6/4.8	80	63	1.56	1.15/2.35/3.55/4.75	80	63
Flip angle (°)	15	60	90	90	12	60	90	90
FOV (cm)	42 × 30	38 × 30	38 × 30	38 × 30	42 × 30	38 × 30	38 × 38	38 × 30
Matrix	320 × 224	288 × 230	512 × 256	128 × 128	304 × 250	288 × 230	304 × 304	128 × 128
Parallel imaging factor	1.5	2.2	2	2	1.7	2	2.5	2
Slice thickness (mm)	4	5	5	6	4	5	5	6
Intersection gap (mm)	− 2	1	1	0	− 2	1	1	0
No. of sections	90	30	30	30	90	30	30	30
Acquisition time	20 s	21 s	3 min	1 min 45 s	20 s	17 s	3 min	1 min 45 s
*b* values (mm^2^/s)	NA	NA	NA	0, 200, 800	NA	NA	NA	0, 200, 800

T1WI, T1-weighted imaging; T2WI, T2-weighted imaging; DWI, diffusion-weighted imaging; 3D, three dimensional; 2D, two dimensional; FFE, fast field-echo; GRE, gradient recalled-echo; TSE, turbo spin-echo; EP, echo-planar; MV, Multi Vane; BH, breath-hold; RT, respiratory-triggered; FB, free-breathing; TR, repetition time; TE, echo time; FOV, field of view; NA, not applicable

Gadolinium-enhanced MRI was performed using T1-weighted fat-suppressed sequence (TR/TE, 3.3/1.55 ms; flip angle, 12°; section thickness, 4 mm; slice gap, -2 mm; field of view, 42 × 30 cm; matrix, 304 × 304; and slice number, 90 slices). The patients received one of the following contrast agents: 0.025 mmol gadolinium (Gd)/kg body weight for Gd-EOB-DTPA (0.25 mmol Gd/mL, Primovist®, Bayer HealthCare) or 0.1 mmol Gd/kg body weight for Gd-DTPA (0.5 mmol Gd/mL, Magnevist®, Bayer HealthCare), Gd-BT-DO3A (1.0 mmol Gd/mL, Gadovist®, Bayer HealthCare), Gd-DOTA (0.5 mmol Gd/mL, Magnescope®[Japan], Dotarem®[US], Guerbet), or Gd-DTPA-BMA (0.5 mmol Gd/mL, Omniscan®, GE Healthcare) were administered. Intravenous contrast material administration was performed at a rate of 2 mL/s followed by a 30 mL saline flush at the same rate. Bolus-tracking method was used for performing the multiphasic pancreatic protocol MRI. The arterial dominant and portal venous phases were obtained 10 and 45 s after the detection of contrast in the abdominal aorta, and late-dynamic phases were obtained at 120 s and 180 s after the administration of the contrast material.

### Image analysis

Two radiologists (Y.N. and N.K., with 10 and 9 years of post-training experience in interpreting abdominal MR images), who were blinded to the clinical information of the patients, reviewed the MR images independently, and then in consensus. Using a commercially available digital imaging software and a Communications in Medicine viewer (ShadeQuest ViewR; Yokogawa Medical Solutions, Tokyo, Japan), which was programmed to perform histogram analyses, the radiologists reviewed the ADC maps. Using a circular region-of-interest (ROI) cursor drawn to encompass as much of the lesion as possible, the radiologists obtained HA parameters on the ADC map with the greatest dimension of PDAC. The ROI placement was performed to include the entire tumors including cystic or necrotic components while carefully avoiding artifacts and flow voids. To determine the precise location of PDAC to enable accurate ROI measurements on the ADC maps, the radiologists were allowed to refer to other MRI sequences. A series of HA parameters were estimated, including mean, standard error, median, mode, standard deviation, variance, kurtosis, skewness, coefficient of variance, minimum, maximum, entropy, and energy.

### Pathological evaluation

Two experienced pathologists (A.H. and H.T., with 33 and 21 years of experience in tumor pathology, respectively) reviewed the pathological specimens obtained at surgery in consensus. TNM staging was performed according to the eighth edition of the American Joint Committee on Cancer staging system [18] and R classification for tumor differentiation was performed according to the Union for International Cancer Control [19]. Hematoxylin and eosin-stained specimens were used for the pathological evaluation. Intratumoral necrosis was defined based on its occurrence within the tumor including cancer cells and stroma. For this study, we defined MITN when coagulation necrosis was fully developed according to a previous report [17]. The pathologists evaluated the presence or absence of MITN for each patient.

### Statistical analysis

Patients’ demographics and tumor characteristics were summarized using frequencies for categorical variables and mean ± standard deviation (SD) with range for continuous variables. Risk factors associated with OS (from surgery to death) were assessed by Cox proportional hazard model with adjustment for age. One multivariable model includes only two parameters to avoid overfitting, that is, one factor and age. In other words, there are 22 Cox models (9 for patients’ demographics and tumor characteristics, 13 for HA parameters) adjusted for age. Time-dependent receiver operating characteristic (ROC) curve [20] was used to assess the predictive performance of the risk factors strongly associated with OS according to the results using a Cox proportional hazard analysis. The bootstrap bias-corrected area under the ROC curve (AUC) was reported as the measure of the predictive performance of the risk factor. Ten thousand of bootstrap samples were generated and the AUCs obtained from each ROC were averaged to calculate the bootstrap AUC-ROC. An appropriate threshold for predicting mortality was also calculated by averaging the thresholds obtained from each ROC. Cumulative survival rates were estimated using the Kaplan–Meier method for each group separated by the threshold. Differences in survival rates between groups defined by the threshold were confirmed by the log-rank test. To assess the relationship between the risk factor and MITN, logistic regression analysis was performed adjusting for age. All *P* values were two-sided. *P* values of < 0.05 were considered statistically significant. All statistical analyses were performed using R version 4.0.2 (www.r-project.org).

## Results

### Patients’ demographics and tumor characteristics

Patients’ demographics and tumor characteristics are summarized in Table 2. The mean body mass index of the patients was 20.7 ± 3.5 kg/m^2^. Plasmatic CEA and CA 19–9 levels were 4.2 ± 3.7 ng/mL and 285.7 ± 404.7 U/mL, respectively. PDACs were located in the pancreatic head (*n* = 21), body (*n* = 11), and tail (*n* = 7). The mean tumor size was 18.7 ± 5.3 mm. Pathological T classification was 0 in 1 patient, 1c in 9 patients, 2 in 26 patients, and 3 in 3 patients. Pathological N classification was 0 in 15 patients, 1 in 13 patients, and 2 in 11 patients. R0 resection was achieved in 33 (84.6%). MITN was observed in 11 patients (28.2%).

**Table 2:** Patients’ demographics and tumor characteristics

Characteristics	*n* = 39
**Patients’ demographics**	
Age (years)	73.3 ± 7.1 (59–84)
Gender (Male:Female)	18 (46):21 (54)
Body mass index (kg/m^2^)	20.7 ± 3.5 (14.9–31.5)
CEA (ng/mL)	4.2 ± 3.7 (0.9–20.2)
CA19-9 (U/mL)	285.7 ± 404.7 (0.1–1818.8)
**Tumor characteristics**	
Location (head/body/tail)	21/11/7
Tumor size (mm)	18.7 ± 5.3 (9.2–30.6)
pT stage (0/1a/1b/1c/2/3/4)	1/0/0/9/26/3/0
pN stage (0/1/2)	15/13/11
TNM stage (1A/1B/2A/2B/3/4)	6/9/0/13/11/0
Tumor differentiation (wel/mod/por)	8/29/2
R classification (0/1)	33/6
Massive intratumoral necrosis (−/ +)	28/11

Data are means ± 1 standard deviation with ranges in parentheses

CEA, carcinoembryonic antigen; CA19-9, carbohydrate antigen 19-9

### Analysis of prognostic factors for OS

The median follow-up duration was 15.3 months (range 3.1–47.9). Median OS was 19.9 months, and 23 (59.0%) patients were alive at the last follow-up for data collection. At 1 and 3 years, the estimated OS rates were 84.2% and 45.9%, respectively.

**Fig. 2:**
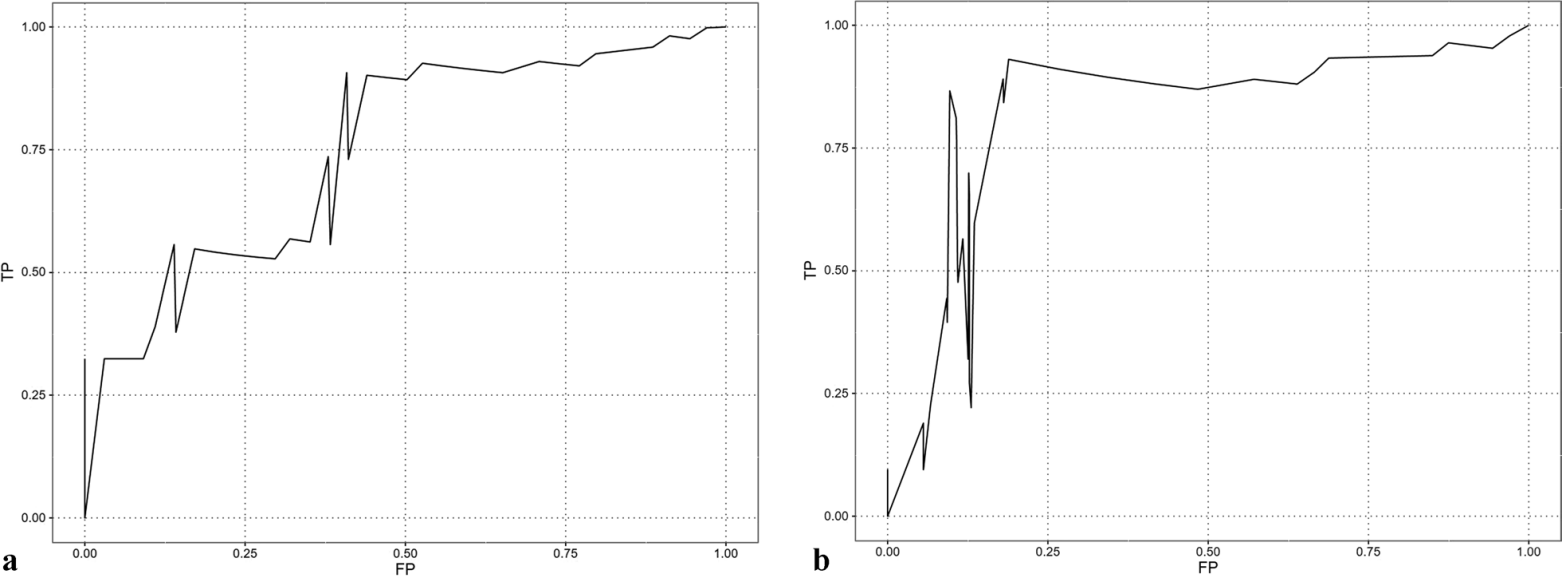
Time-dependent receiver operating characteristic (ROC) curves for predicting **a** 1- and **b** 3-year survival. The bootstrap area under the ROC curves for predicting 1- and 3-year survival were 0.755 and 0.824 when using cutoff values of 2.83 and 2.45, respectively

**Fig. 3:**
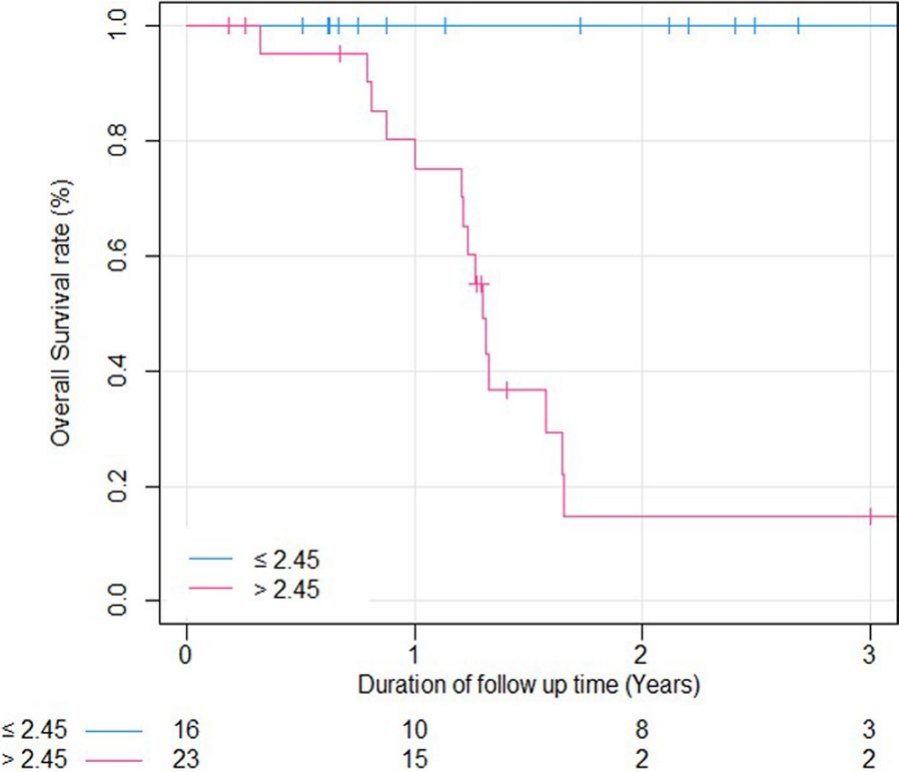
Kaplan–Meier 3-year survival curves for PDACs with high and low kurtosis values. Patients with high kurtosis exhibited lower survival rates than those with low kurtosis (*P* < 0.001: 1-year survival rate, 75.2% vs. 100%: 3-year survival rate, 14.7% vs. 100%)

The results of the multivariable Cox proportional hazard analysis of prognostic factors for OS are shown in Table 3. The significant prognostic factors for OS from patients’ demographics and tumor characteristics were tumor location (hazard ratio [HR] [95% lower confidence level and 95% upper confidence level], 1.00 in the pancreatic head, 0.05 [0.01, 0.37] in the pancreatic body, and 0.17 [0.04, 0.70] in the pancreatic tail; *P* = 0.003), pathological N stage (HR [95% lower confidence level and 95% upper confidence level], 1.00 in pN0, 2.23 [0.59, 8.48] in pN1, and 5.4 [1.46, 20.00] in pN2; *P* = 0.038), R classification (HR [95% lower confidence level and 95% upper confidence level], 1.00 in R0 and 4.14 [1.42, 12.07] in R1; *P* = 0.009), whereas those from HA parameters were kurtosis (HR for the 75th vs 25th percentile [95% lower confidence level and 95% upper confidence level], 7.11 [2.42, 20.90]; *P* < 0.001), entropy (HR for the 75th vs 25th percentile [95% lower confidence level and 95% upper confidence level], 2.06 [1.16, 3.66]; *P* = 0.013), and energy (HR for the 75th vs 25th percentile [95% lower confidence level and 95% upper confidence level], 0.49 [0.25, 0.97]; *P* = 0.04). Kurtosis had the strongest association with OS among these prognostic factors; therefore, time-dependent ROC curve and bootstrap AUC analyses were conducted for the kurtosis. The cutoff values of the kurtosis for predicting 1- and 3-year survival were 2.83 and 2.45 based on bootstrap AUC. The bootstrap AUCs for predicting 1- and 3-year survival were 0.755 and 0.824 when using these cutoff values (Fig. 2). Patients with high kurtosis (greater than cutoff value) exhibited lower survival rates than those with low kurtosis (cutoff value or less) (*P* < 0.001: 1-year survival rate, 75.2% versus 100%: 3-year survival rate, 14.7% versus 100%) (Fig. 3).

**Table 3:** Cox proportional hazard analysis of prognostic factors for overall survival

Parameter	HR	95%LCL	95%UCL	*P* value
**Patients’ demographics and tumor characteristics**				
CEA (ng/mL), IQR 1.70–4.95	1.00	0.92	1.09	0.945
CA19-9 (U/mL), IQR 37.55–333.45	1.34	0.98	1.84	0.067
Tumor location				0.003*
Head	1			
Body	0.05	0.01	0.37	
Tail	0.17	0.04	0.7	
Tumor size (mm)	1.78	0.93	3.42	0.081
pT stage				0.221
0	1			
1c	16.82	0	1.27e + 46	
2	83.65	0	6.21e + 46	
3	206.67	0	1.55e + 47	
pN stage				0.038*
0	1			
1	2.23	0.59	8.48	
2	5.4	1.46	20.0	
TNM stage		0.088		
1A	1			
1B	1.69	0.17	16.4	
2B	3.21	0.37	27.94	
3	7.74	0.92	65.02	
Tumor differentiation				0.775
Poor	1			
Moderate	1.89	0.23	15.5	
Well	1.34	0.11	16.24	
R classification	4.14	1.42	12.07	0.009*
**Histogram analysis parameters**				
Mean, IQR 1320.57–1740.09	0.64	0.32	1.29	0.212
Standard error, IQR 17.84–36.08	0.63	0.32	1.24	0.183
Median, IQR 1318.04–1720.21	0.64	0.62	1.27	0.198
Mode, IQR 1237.17–1586.52	0.88	0.53	1.47	0.629
Standard deviation, IQR 96.20–192.00	1.02	0.56	1.86	0.941
Variance, IQR 9268.55–36,867.38	0.93	0.58	1.51	0.771
Kurtosis, IQR 2.20–3.06	7.11	2.42	20.9	< 0.001*
Skewness, IQR -0.50–0.35	0.92	0.37	2.31	0.86
CV, IQR 0.06–0.13	1.25	0.67	2.33	0.479
Minimum, IQR 1070.98–1412.84	0.62	0.35	1.11	0.107
Maximum, IQR 1626.85–2044.53	0.83	0.5	1.4	0.49
Entropy, IQR 1.29–1.56	2.06	1.16	3.66	0.013*
Energy, IQR 0.03–0.05	0.49	0.25	0.97	0.04*

HR for continuous variable represents the risk of the 75th percentile relative to the 25th percentile. HR for categorical variable is set to 1 for the reference category

e + 46 means 10 to the 46th power. e + 47 means 10 to the 47th power

HR, hazard ratio; LCL, lower confidence limit; UCL, upper confidence limit; IQR, interquartile range (25th-75th percentile); CV, coefficient of variance

*P < 0.05, significant difference

### Correlation of the HA parameters with massive intratumoral necrosis

**Fig. 4:**
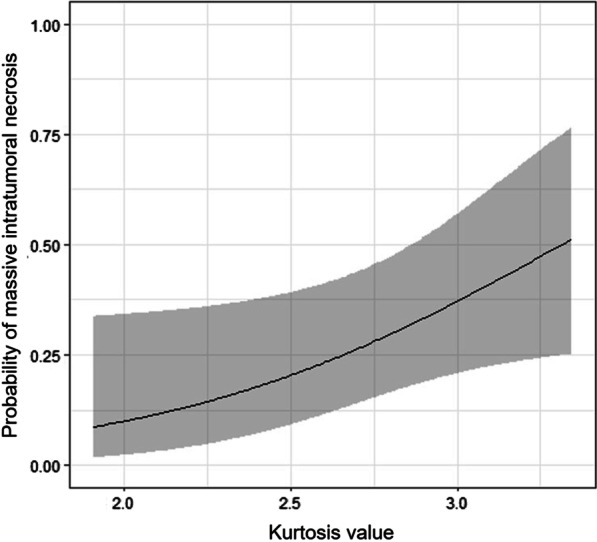
The relationship between the kurtosis of tumor ADC values and massive intratumoral necrosis (MITN). Logistic regression analysis with adjustment for age demonstrated that the kurtosis is associated with MITN (odds ratio, 4.20; *P* = 0.045)

**Fig. 5:**
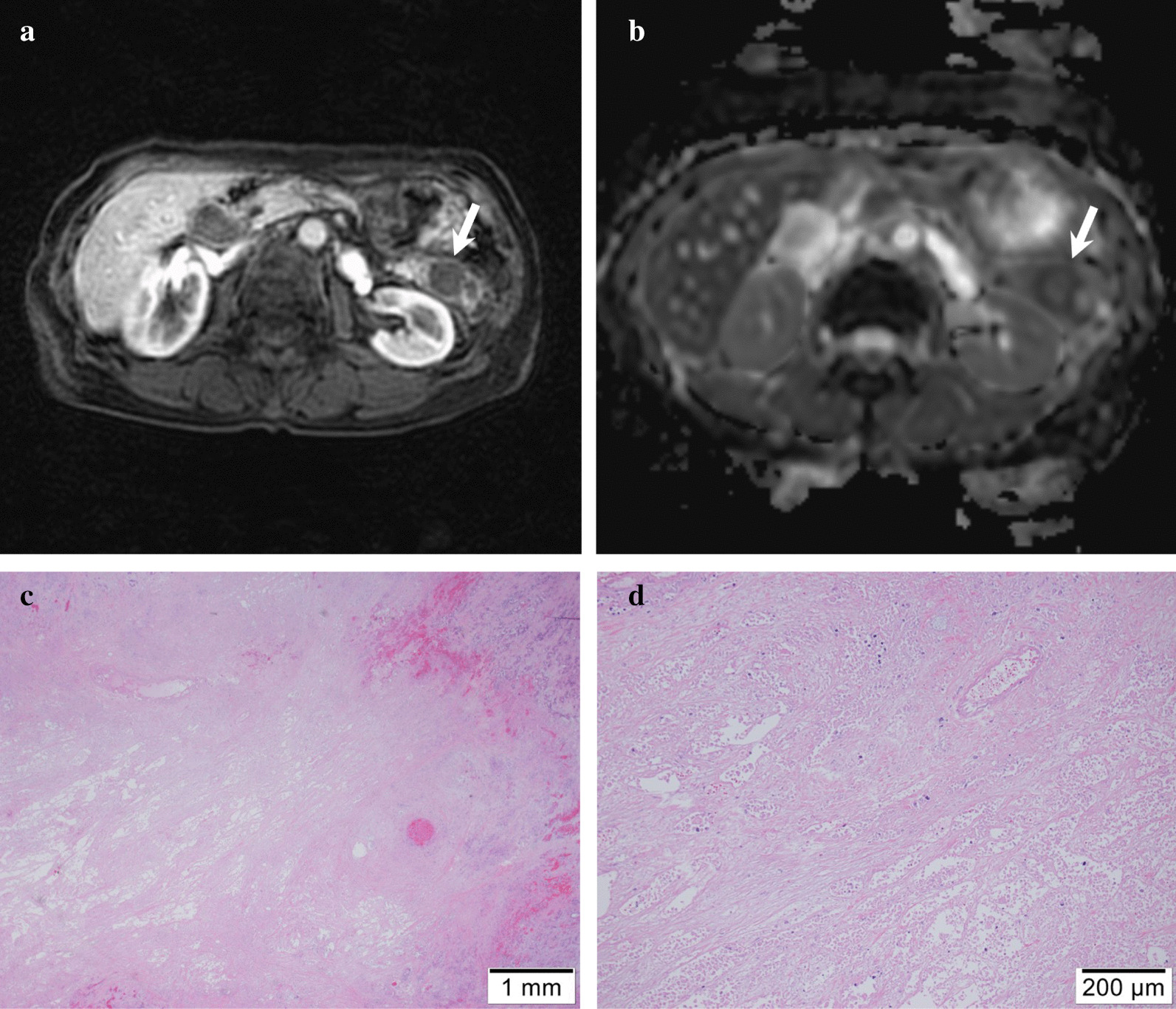
A 74-year-old female with pancreatic ductal adenocarcinoma at the tail. **a** Axial arterial phase image and **b** apparent diffusion coefficient (ADC) map show pancreatic mass with restricted diffusion at pancreatic tail (arrow). The kurtosis of tumor ADC value was 3.1. **c**, **d** Microphotograph of hematoxylin and eosin-staining for PDAC revealing the massive intratumoral necrosis

**Fig. 6:**
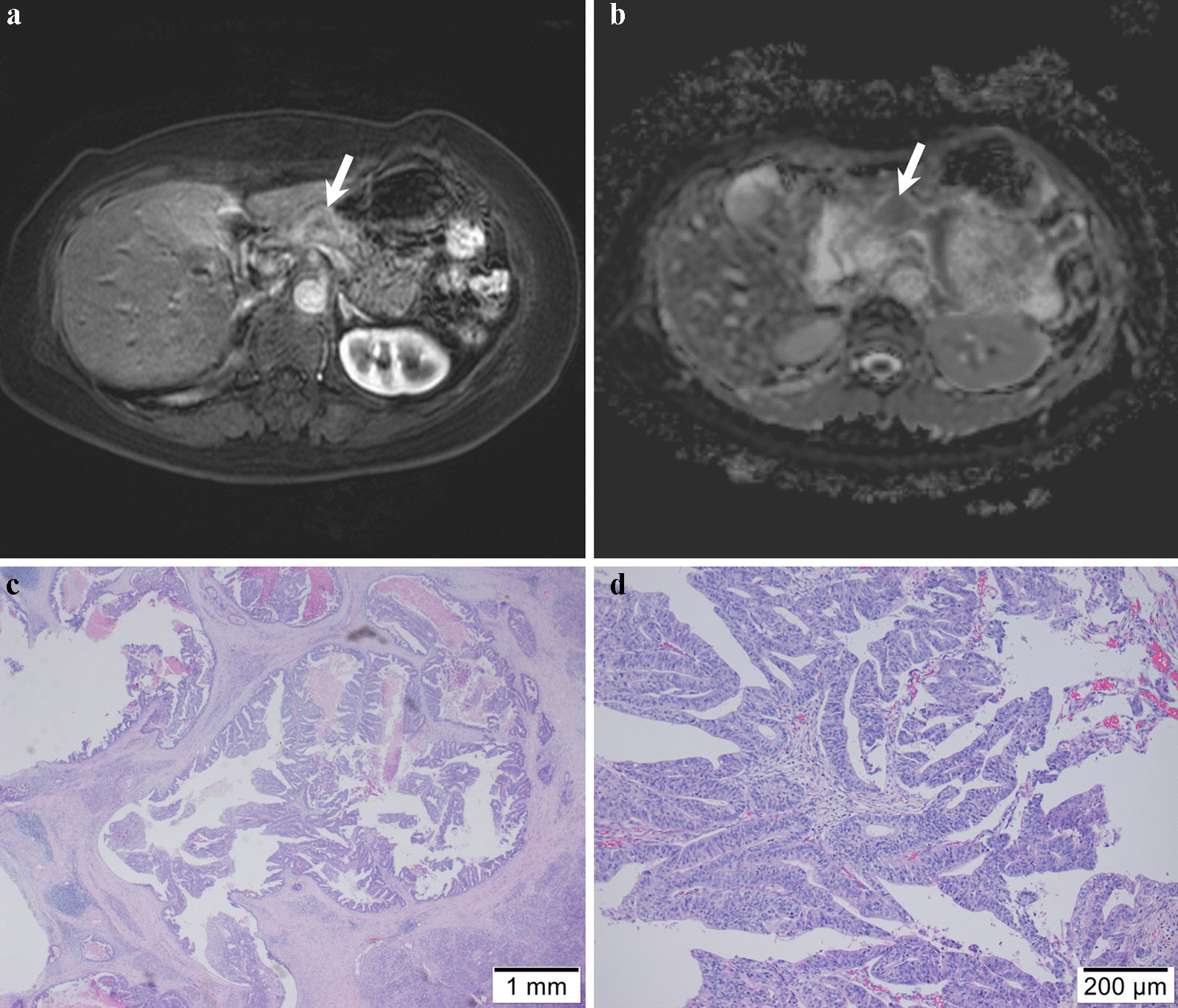
A 63-year-old female with pancreatic ductal adenocarcinoma at the body. **a** Axial arterial phase image and **b** apparent diffusion coefficient (ADC) map show pancreatic mass with restricted diffusion at pancreatic body (arrow). The kurtosis of tumor ADC value was 2.19. **c**, **d** Microphotograph of hematoxylin and eosin-staining for PDAC revealing the no massive intratumoral necrosis

Logistic regression analysis with adjustment for age demonstrated that the kurtosis was associated with MITN (odds ratio, 4.20; *P* = 0.045) (Fig. 4). In MITN positive cases, the kurtosis was significantly higher than in negative cases (3.10 vs. 2.41; *P* = 0.023) (Figs. 5 and 6). On the other hand, we found no significant correlation in the entropy (odds ratio 1.54; *P* = 0.28) and energy (odds ratio 0.73; *P* = 0.37) with MITN.

## Discussion

Our study demonstrated that the kurtosis of tumor ADC values obtained from preoperative MR images is an independent prognostic factor for OS in patients with PDAC. Pathological N stage or R classification were also significant prognostic factors in multivariable Cox proportional hazard analysis; however, these factors are not applicable in the preoperative setting.

HA is a mathematical technique that can evaluate the distribution of gray-level tones on biomedical images and reflects the frequency of pixels exhibiting gray levels that lie within a given interval [21]. HA parameters could reflect information within tumors that cannot be assessed with conventional imaging, including tumor heterogeneity such as necrosis that may represent tumor aggressiveness and prognostic implication [21]. Positive kurtosis means a sharper peak and wider tails. We infer that the reason for patients with high kurtosis exhibiting lower survival rates than those with low kurtosis was due to its association with the heterogeneity of PDAC and presence of hypovascular or hypoxic tissues, and MITN. Hypoxia has been associated with angiogenesis, tumor aggressiveness, and poor prognosis. Generally, the central portion becomes hypoxic, hypo-vascular, and necrotic as the tumor grows [22]. It is believed that these tumors, having MITN, contain large necrotic area in the central portion and a few viable tumor cells in the periphery of the tumor. Therefore, we believe that these tumors show high kurtosis of tumor ADC values. The kurtosis of tumor ADC values was significantly higher in MITN positive cases than in negative cases, as shown in this study, and the kurtosis of tumor ADC values was significantly associated with MITN. In the Cox proportional hazard analysis, entropy and energy showed a significant association with OS as well; however, these parameters were not correlated with MITN. Tumor necrosis has been reported as a prognostic factor in patients with PDAC [14, 16, 17]; however, the presence or absence of tumor necrosis is not preoperatively assessed. We believe that the kurtosis of tumor ADC values could indicate the presence of MITN and could be a prognostic factor in patients with PDAC.

The imaging findings associated with MITN and poor prognosis, including a hypodense tumor in the portal venous phase CT [14] and rim enhancement at dynamic contrast-enhanced MRI [16], were reported in previous studies. Occurrence of MITN in the central portion of PADC reflects a relatively low density/signal intensity area with poor enhancement on dynamic contrast-enhanced imaging and the viable tumor cells in the periphery of the tumor. While these imaging findings are valuable in predicting patients' outcome, these are subjective imaging appearances. On the contrary, kurtosis of tumor ADC values is an objective and quantitative metric. In 2007, the Quantitative Imaging Biomarker Alliance was established by the Radiological Society of North America to improve the value and practicality of quantitative imaging biomarkers by reducing variability across devices, patients, and time [23]. Our results demonstrate that kurtosis obtained from HA is a quantitative imaging biomarker with profound significance in predicting patients' OS.

Our study had several limitations. First, this was a retrospective study performed at a single center with a small sample size, which may result in selection bias. The external validation dataset lacks to verify the threshold of kurtosis of tumor ADC values found in this study. Second, to avoid overfitting of the model used in the analysis, we could not consider the removal of biases other than age in confirming the association between OS and predictors. Third, we included both patients who underwent neoadjuvant chemotherapy (*n* = 12) and surgical resection without neoadjuvant chemotherapy (*n* = 27). HA and pathological evaluation were performed after neoadjuvant chemotherapy in patients who underwent neoadjuvant chemotherapy. It is perceivable that neoadjuvant chemotherapy caused more MITN compared to patients without neoadjuvant chemotherapy. Our HA and pathological results might be affected by neoadjuvant chemotherapy. Finally, we used various MRI scanners because the study period was relatively long. This might have affected the values of HA parameters; nevertheless, the imaging protocols were standardized. Therefore, further clinical investigations in larger patient cohort and a homogeneous patient population are needed to validate our results.

## Conclusion

In conclusion, the kurtosis of tumor ADC values obtained from HA was associated with MITN and prognosis in patients with PDAC. Therefore, this parameter could be used as a quantitative imaging biomarker for predicting OS.

## Data Availability

All data generated or analyzed during this study are included in this published article.

## Abbreviations

ADC: Apparent diffusion coefficient
DWI: Diffusion-weighted imaging
MITN: Massive intratumoral necrosis
MRI: Magnetic resonance imaging
OS: Overall survival
PDAC: Pancreatic ductal adenocarcinoma
HA: Histogram analysis
